# A retrospective analysis of *P. falciparum* drug resistance markers detects an early (2016/17) high prevalence of the k13 C469Y mutation in asymptomatic infections in Northern Uganda

**DOI:** 10.1128/aac.01576-23

**Published:** 2024-08-13

**Authors:** Rodney Ogwang, Victor Osoti, Kevin Wamae, Leonard Ndwiga, Kelvin Muteru, Albert Ningwa, James Tuju, Sam Kinyanjui, Faith Osier, Kevin Marsh, Philip Bejon, Richard Idro, Lynette Isabella Ochola-Oyier

**Affiliations:** 1 Centre for Geographic Medicine Research (Coast), Kenya Medical Research Institute-Wellcome Trust Research Programme, Kilifi, Kenya; 2 Makerere University College of Health Sciences, Kampala, Uganda; 3 Centre of Tropical Neuroscience (CTN), Kitgum Site, Uganda; 4 Department of Life Sciences, Imperial College London, London, United Kingdom; 5 Centre for Tropical Medicine and Global Health, Nuffield Department of Medicine, University of Oxford, Oxford, United Kingdom; The Children's Hospital of Philadelphia, Philadelphia, Pennsylvania, USA

**Keywords:** *Plasmodium falciparum*, molecular surveillance, amplicon sequencing, artemisinin resistance

## Abstract

The emergence of drug-resistant *Plasmodium falciparum* parasites in sub-Saharan Africa will substantially challenge malaria control. Here, we evaluated the frequency of common drug resistance markers among adolescents from Northern Uganda with asymptomatic infections. We used an established amplicon deep sequencing strategy to screen dried blood spot samples collected from 2016 to 2017 during a reported malaria epidemic within the districts of Kitgum and Pader in Northern Uganda. We screened single-nucleotide polymorphisms within: kelch13 (*Pfk13*), dihydropteroate synthase (*Pfdhps*), multidrug resistance-1 (*Pfmdr1*), dihydrofolate reductase (*Pfdhfr*), and apical membrane antigen (*Pfama1*) genes. Within the study population, the median age was 15 years (14.3–15.0, 95% CI), and 54.9% (78/142) were *Plasmodium* positive by 18S rRNA qPCR, which were subsequently targeted for sequencing analysis. We observed a high frequency of resistance markers particularly for sulfadoxine-pyrimethamine (SP), with no wild-type-only parasites observed for *Pfdhfr* (N51I, C59R, and S108N) and *Pfdhps* (A437G and K540E) mutations. Within *Pfmdr1,* mixed infections were common for NF/NY (98.5%). While for artemisinin resistance, in kelch13, there was a high frequency of C469Y (34%). Using the pattern for *Pfama1,* we found a high level of polygenomic infections with all individuals presenting with complexity of infection greater than 2 with a median of 6.9. The high frequency of the quintuple SP drug-resistant parasites and the C469Y artemisinin resistance-associated mutation in asymptomatic individuals suggests an earlier high prevalence than previously reported from symptomatic malaria surveillance studies (in 2016/2017). Our data demonstrate the urgency for routine genomic surveillance programs throughout Africa and the value of deep sequencing.

## INTRODUCTION

Globally, *Plasmodium falciparum* is a leading cause of morbidity and mortality, particularly in sub-Saharan Africa (1, 2). The burden of malaria has steadily declined over the past decades with extensive application of control measures (3). However, more recently, progress appears to have stalled (4), and the WHO reports an estimated 249 million cases with 608,000 malaria deaths in 2022 (2). A major concern is the emergence of artemisinin drug-resistant parasites (5–7). Malaria treatment, particularly with artemisinin-based combination therapies (ACTs), has been a cornerstone of malaria control (8, 9). In most of sub-Saharan Africa, the first-line treatment for uncomplicated malaria is artemether-lumefantrine (AL) or artesunate/amodiaquine, while IV artesunate is used for severe malaria (10). Molecular genetic surveillance can play an important role in tracking the spread of drug-resistant parasites (11–13). This approach may guide the judicious application of available control interventions, potentially halting the spread of resistant parasites (12).

Previously, chloroquine and sulfadoxine-pyrimethamine (SP) resistance arose first in southeast Asia and spread later to Africa (14, 15). Due to the use of SP in intermittent preventive treatment in pregnancy, it is still important to monitor the resistance-conferring genes, *P. falciparum* dihydrofolate reductase (*Pfdhfr*) and dihydropteroate synthase (*Pfdhps*). Similarly, over the past 10 years, artemisinin-resistant parasites have emerged and spread in the Greater Mekong sub-region of southeast Asia and now appear to be emerging in sub-Saharan Africa (15–20). Furthermore, in the *P. falciparum* multidrug resistance 1 (*Pfmdr1*) gene, linkage has been observed between N86 (wild type) and reduced susceptibility to lumefantrine, which is widely used as the artemisinin partner drug in East Africa (21). It is important to note that the increased risk for AL resistance selection is a result of the current high and historical use of AL. In Africa, AL accounts for 85% for all ACTs procured for Africa and indeed Uganda.

Mutations in the propeller domain of the kelch (K13) gene remain the primary molecular markers for artemisinin resistance (22). Although genetic surveillance studies across Africa have detected various mutations associated with resistance, only recently were parasites associated with delayed artemisinin parasite clearance identified in clinical studies from Rwanda (19) and Uganda (18). Important to this report is the study in North Uganda between 2015 and 2019, which assessed the frequencies of two mutations C469Y and A675V using symptomatic samples from a health facility in Gulu district, showing a rising (from about 4% to 20%) frequency of these mutations since 2018 and 2016, respectively (18). Therefore, evaluating the frequency of genetic resistance markers within different populations remains vital (11).

In malaria endemic regions, such as in sub-Saharan Africa, mixed infections are common (23). In a study of 15 countries across Africa, about 55% of the 2,263 whole-genome sequenced *P. falciparum* isolates were polygenomic with up to 9 clones in an infection. High malaria transmission drives high parasite mixing and a high complexity of infection (COI), thus mixed infections are likely key to the genesis and transmission of new mutations within a population. Notably, a high inbreeding coefficient (Fws) was observed in samples from Western Kenya, which is a region of holoendemic malaria transmission (23), a situation most likely maintained within asymptomatic infections that often go undetected by passive case detection surveillance studies in malaria endemic populations.

Here, we screened dried blood spot samples (DBS) collected from children and adolescents with asymptomatic infections from two districts, Kitgum and Pader, in Northern Uganda during a period of heightened malaria transmission (2016–2017). A study from Kitgum General Hospital showed a rise in malaria cases and hospitalization, the malaria test positivity rate increased from 10.5% to 54.6% between 2014 and 2016, and similarly, malaria accounted for over 40% of inpatient admissions (24). In malaria endemic areas, asymptomatic infections are common and are a major concern for elimination efforts (25). Patients with asymptomatic infections are typically older with some level of naturally acquired immunity (26). In the absence of symptoms such individuals do not seek care and, in turn, are silent reservoirs perpetuating malaria transmission (27). We aimed to estimate the presence of drug-resistant markers in asymptomatic *P. falciparum* infections using a targeted amplicon deep sequencing (AmpSeq) method. Furthermore, we assessed the level of genetic diversity and COI using the *P. falciparum* apical membrane antigen 1 (*Pfama1*) gene—a highly polymorphic merozoite surface antigen (28).

## RESULTS

### Population demographics and prevalence of asymptomatic infections

The median age of the participants was 15 (14.3–15.0, 95% CI), and 56.3% (80/142) were male. All study participants were healthy with no significant clinical symptoms. A total of 54.9% (78/142) were positive based on the *Pf*18S rRNA qPCR. Among those positive by qPCR, 16.6% (13/78) were negative by *P. falciparum* histidine-rich protein-2 (HRP2) rapid diagnostic tests (RDTs) (CareStart). All positive infections were asymptomatic (confirmed parasitemia in the absence of fever—body temperature below 37.5°C).

### High frequency of SP resistance markers and mdr1 genotypes linked to reduced susceptibility to lumefantrine

Of the 74.4% (58/78) samples successfully genotyped for *Pfdhfr,* high frequencies of mutations for resistance were shown at three loci (N51I, C59R, and S108N), but parasites were largely wild type at codon 164 (Table 1). The double, ICNI, and triple, IRNI, haplotypes were observed in 33% and 51% of the samples, respectively, while infections containing the quadruple mutant, IRNL were at 7%. However, the adjusted microhaplotype frequencies weighted by the within-host frequencies were 11%, 88%, and 0.7%, respectively, demonstrating the dominance of the triple mutant, IRNI (Table S1). For the *Pfdhps* gene, 91% (71/78) were successfully genotyped. The mutations A437G and K540E, with the inclusion of the mixed genotypes, were at high frequency (100%) and since there were no wild-type-only infection (Table 1). However, S436A, S436C, P568L, G579E, and A581G mutants were at low frequency with most parasites being wild type for these loci (Table 1). The double SGEA haplotype was observed at 35.5% and single mutants SGEA (35.5%), SGKA (30.5%), and SAEA (12%). The wild-type (SAKA) haplotype was observed at 13.5%, while the triple mutant (SGEG) was less common and observed in less than 5% of samples (Table 2). The adjusted frequencies were 82% for SGEA and <10% for all other microhaplotypes (Table S1).

**TABLE 1:** Frequency of mutations from each drug-resistant gene in the population^*b*^

Gene	Mutation	No. successfully sequenced	Frequency (%)		
			Wild type	Mutant	Mixed
*dhfr*	N51I	58	0	82.8	17.2
	C59R		0	36.2	63.8
	S108N		0	98.3	1.7
	I164L		86.2	0	13.8
*dhps*	S436/A/C^*a*^	71	93	0	7
	A437G		0	54.9	45.1
	K540E		0	9.9	90.1
	P568L		90.1	0	9.9
	G579E		98.6	0	1.4
	A581G		90.1	0	9.9
*k13*	C469Y	50	66.0	0	34.0
	E507G		96	0	4
	A578S		96.0	0	4.0
*mdr1*	N86Y	67	100	0	0
	Y184F		0	1.5	98.5
	T199S		68.7	0	31.3

^*a*^ S436A was at 2.8%, while S436C was at 4.2%.

^*b*^ Mixed indicates infections with both the mutant and the wild-type allele. *k13* codons 446, 458, 476, 477, 493, 539, 543, 553, 561, and 580 were 100% wild-type *dhfr*.

**TABLE 2:** Frequency of resistance haplotypes for each gene and of infections containing mixed haplotypes^*b*^

Gene (codon)	Haplotype	n	Frequency (%)
*dhfr* (51, 59, 108, 164)	**IRN**I	58	51.3
	**I**C**N**I	37	32.7
	N**RN**I	9	7.9
	**IRNL**	8	7.1
	NCSI^*a*^	1	0.9
*dhps* (436, 437, 540, 581)	S**GE**A	71	35.5
	S**G**KA	61	30.5
	SAKA^*a*^	27	13.5
	SA**E**A	24	12.0
	S**GEG**	7	3.5
	**C**A**E**A	3	1.5
	**C**AKA	3	1.5
	**A**A**E**A	2	1.0
*k13* (469, 482, 507, 578)	CYEA^*a*^	50	68.5
	**Y**YEA	17	23.3
	CYE**S**	2	2.7
	C**H**EA	2	2.7
	**Y**Y**G**A	2	2.7
*mdr1* (86, 184, 199)	N**F**T	67	44.1
	NYT^*a*^	64	42.1
	NY**S**	21	13.8

^*a*^ Wild type, bold text indicates the mutant codon. The *k13* 482 H and 507G mutants were observed in two samples.

^*b*^ The number of samples genotyped (*dhfr* = 58, *dhps* = 71, *k13* = 50 while *mdr1* = 67 samples). The frequency percentage of each haplotype is determined by the number of occurrences of a particular haplotype divided by the total number of haplotypes, denoted as “*n*”.

For the *Pfmdr1* gene, NFT haplotype was observed at high frequency (44.1%), while the wild-type NYT haplotype was observed at 42%, as mixed infections in the population. Their adjusted frequencies were 59% and 37%, respectively (Table S1). The newly described haplotype (NYS) was observed at 13.8% frequency (Table 2), with a much lower prevalence of 4% (Table S1).

### A high frequency of artemisinin resistance markers

*Pfkelch13* propeller domain genotyping was successful from 50 samples. Two *Pfkelch13* mutations (A578S and C469Y) were detected at 4% and 34%, respectively. Surprisingly, we observed the presence of both the C469Y and rare E507G mutations in two samples.

### Asymptomatic individuals present high levels of complexity of infection

**Fig 1:**
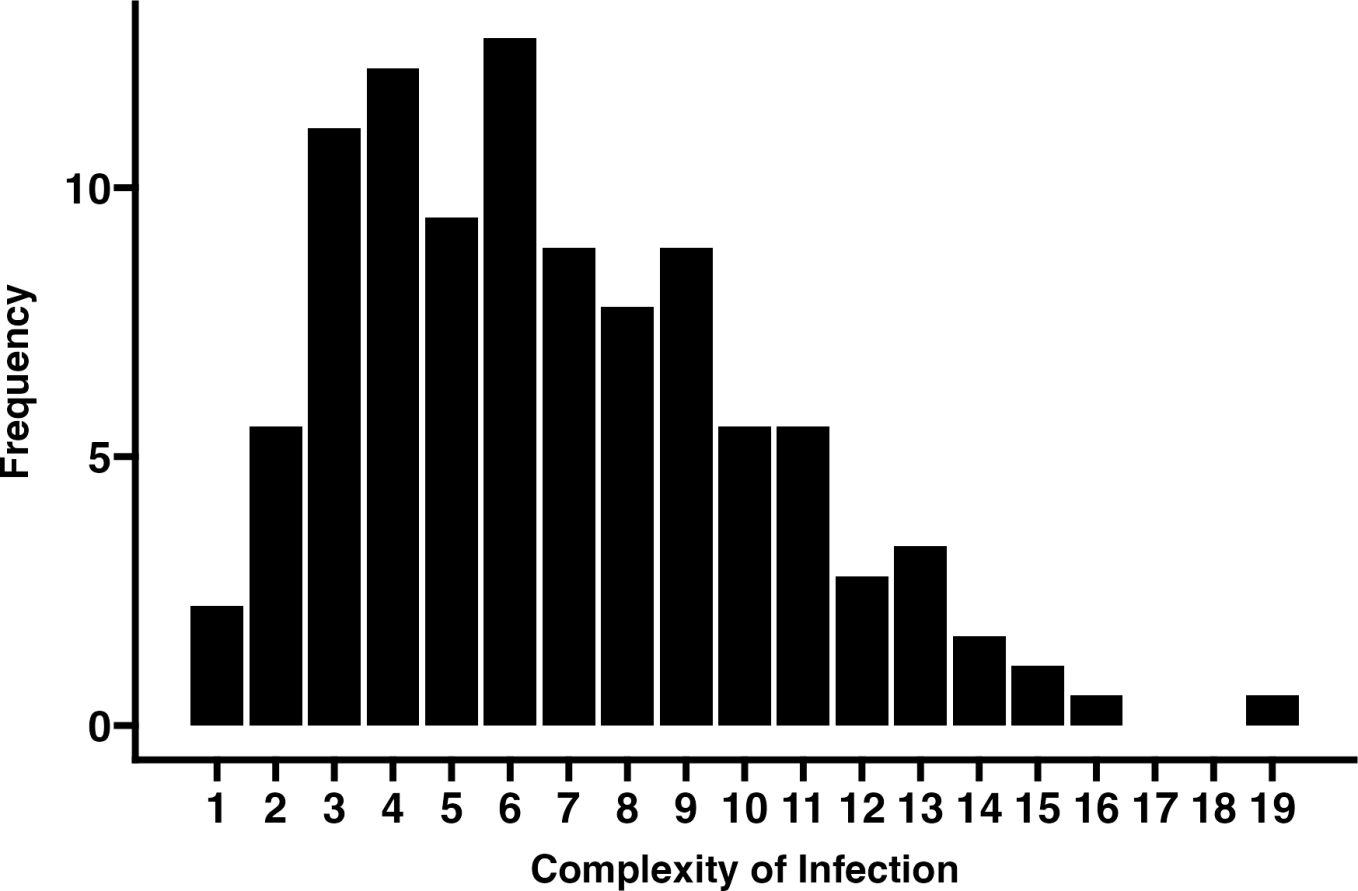
Distribution of individuals with genetically distinct parasite infections using Pf-AMA1 genotyping. The y-axis is the frequency of individuals, and on the x-axis is the complexity of infections.

The highly polymorphic *Pfama1* gene was used to investigate the complexity of infection. Among the 78 samples positive by the 18S rRNA qPCR, 70 samples (90%) were successfully sequenced. A total of 74 unique haplotypes were identified, and all 70 individuals harbored polyclonal infections (COI >2). The mean COI was 6.97 (range 2–19) clones per individual (Fig. 1). *Pfama1* showed more evenly distributed combinations where minor alleles were frequently present without mixture. Some *Pfama1* loci contained all three combinations per locus of wild type, mutant, and mixed, e.g., codons 200, 204, 225, 230, 242, 243, 267, 283, and 285 (Table 3).

**TABLE 3:** Frequency of mutations from the Pfama1 gene in the population^*a*^

Mutation	Frequency (%)		
	Wild type	Mutant	Mixed
N162K	28.4	0	71.6
T167K	0	38.8	61.2
G172E	0	4.5	95.5
N173K	64.2	0	35.8
Y175D	17.9	0	82.1
E187N/K/I	0	1.5	98.5
L189P/H	17.9	0	82.1
M190I	0	3.0	97.0
D196N	10.4	0	89.6
E197G/Q/D/H/R	0	0	100
R199K	53.7	0	46.3
H200D/L/R	10.4	1.5	88.1
F201L/S	7.5	0	92.5
K203E	98.5	0	1.5
D204N/K	5.9	7.5	86.6
K206E	20.9	1.5	77.6
Y207D	16.4	3	80.6
M224I	100	0	0
I225N	13.4	1.5	85.1
D227Y	80.6	0	19.4
N228K/I	47.8	0	52.2
K230Q	6.0	1.5	92.5
D242Y	3.0	1.5	95.5
K243E/N	0	11.9	88.1
D244N/Y	50.7	0	49.3
K245N	37.3	0	62.7
E267Q	1.5	4.5	94.0
K269I	25.4	0	74.6
I282K/E	11.9	0	88.1
S283L	11.9	1.5	86.6
Q285E	16.4	1.5	82.1
N286D	97	0	3.0
K292	100	0	0
D296H	32.8	0	67.2
E299K	92.5	0	7.5
K300E	19.4	0	80.6

^*a*^ Total number of samples sequenced = 78.

## DISCUSSION

The drug resistance markers were primarily mixed genotype infections with double, triple, and quadruple *Pfdhfr* mutants, demonstrating the extent of mutations within individual asymptomatic infections. Of note, quadruple *Pfdhfr* mutations, the inclusion of codon I164L, and *Pfdhps* A581G mutations were present but at much lower frequencies of <15%. They remain important loci to monitor and determine the extent of high levels of SP resistance. As expected, the triple *Pfdhfr* and double *Pfdhps* mutations continue to be maintained at high frequencies (29, 30). Despite the high levels of genetic resistance markers to SP, this drug is still recommended for intermittent preventive treatment in pregnancy (IPTp) in malaria-endemic areas of Africa (31). Recent evidence demonstrates that the impact of the chemoprophylactic use of SP in pregnancy may not be through the prevention of malaria infection specifically, but it appears to be associated with improved birth outcomes, i.e., a reduction in the risk of low birth weight (32) .

Studies conducted around the districts of Gulu, Lamwo, and Agago, which are all within 100 km of both Pader and Kitgum (the community sites for this study), described an increasing presence of the C469Y mutation in about 40 symptomatic cases at a prevalence of about 2% in 2017 (18) to >15% in 2019 (33) in Northern Uganda. A more recent report suggests stable circulation at approximately 20% prevalence of these mutations in 2022 among uncomplicated infections (34). These concerning findings highlight the high prevalence of the C469Y mutation beyond the routine detection of the easily identifiable malaria cases due to the presence of symptoms, while the reservoir of malaria transmission continues to go undetected, even though they make up a large percentage of malaria infections in malaria-endemic regions. In Pader and Kitgum, in 2016/17, asymptomatic RDT-positive malaria infections were at a prevalence of 55%. Furthermore, the independent emergence and local spread of clinically artemisinin-resistant *P. falciparum* have recently been documented in Africa (2, 5). In Uganda, countrywide studies done from 2016 to 2019 suggested that these alleles were restricted to the northern half of the country (30). Recently, the C469Y mutation was observed at a frequency of 34% in Northern Uganda compared to 3% in Eastern Uganda. Furthermore, isolates with this mutation were significantly less susceptible to lumefantrine, even in mixed, C469, and 469Y infections (35). Perhaps, the presence of the C469Y and A675V [a mutation described by Balikagala and colleagues (18)] may have been a factor that reduced ACT drug efficacy in the districts of Kitgum and Pader in Northern Uganda during the period of heightened transmission (24, 36, 37). This artemisinin resistance mutation emphasizes the need for continuous, longitudinal genomic surveillance for the early detection and monitoring of drug resistance. This study further strengthens the requirement of active genomic surveillance endeavors in the community (12, 38). This could allow for the early detection of important resistant mutations (18, 39).

The asymptomatic infections assessed in Pader and Kitgum were primarily polyclonal infections, reflecting the high and perennial malaria transmission in this region of Northern Uganda. This is similar to other reports in high malaria burden areas that have high levels of polyclonal infections and genetic diversity (28, 40). Much of the data on genomic markers of parasite resistance comes from research groups (41) and has been opportunistic, resulting in a substantial delay in the identification of increasing prevalence of drug resistance. Our deep sequencing approach showed a higher-than-expected frequency of mixed infections for these alleles encoding drug resistance, in contrast to the pattern for AMA1, where minor alleles were often present in pure infections. This high frequency of mixed infections not only reflects the high COI in this population but provides an insight into how resistance mutations emerge, particularly in asymptomatic individuals who are not likely to be treated. Infections are initially, purely wild type, present as mixed infections and thereafter purely drug resistance when the prevalence of the mutation is high in the population due to a high selection pressure. The mixed infections would usually be missed by capillary sequencing approaches; the short-fragment deep sequencing approach increases the accuracy in calling haplotypes and mixed infections. Taken together, our data demonstrate the urgent need for the inclusion of asymptomatic infections in programmatic genomic surveillance to guide public health.

Molecular surveillance is an important tool for tracking the prevalence of mutations that may affect the efficacy of anti-malarial drugs (12, 13). Furthermore, multi-strain infections may impact malaria disease management and transmission (40). It has also been proposed that multiclonal malaria infections can influence clinical outcomes (40) and may have a negative impact on an individual’s response to anti-malarial drug treatment (42). Sensitive detection methods, such as amplicon-based deep sequencing, could enable a better understanding of malaria strain diversity in relation to potential malaria vaccine antigen candidates and track changes in parasite genetic diversity (43).

This study had some limitations. The sample size following sequencing was relatively small due to the low parasitemia in asymptomatic infections. However, the detection of these mutations gives an early indication of the presence of the drug resistance mutations in a previously and not routinely sampled population. Furthermore, the analysis of the microhaplotype frequencies by two approaches demonstrates the diversity of the infections based on the number of observations per infection and the frequencies of the microhaplotypes in the population based on a weighted within-host frequency. This study does provide a valuable baseline for future studies within the districts of Kitgum and Pader in Northern Uganda, a region where clinical evidence of prolonged parasite clearance (18) and an increase in the C469Y mutation frequency have been described. Second, this study did not follow up with any individuals and does not demonstrate clinical evidence of resistance. Finally, we did not investigate the prevalence of the A675V mutation associated with artemisinin resistance, which indeed may be circulating at a higher frequency. Studies in districts surrounding the study area estimate prevalence at approximately 41% (33). The greater resolution provided by AmpSeq distinguishes loci under positive selection in the population, such as those of drug resistance markers, that were classified as either mutant and mixed or wild-type and mixed genotypes. In great contrast, for genes under balancing selection, such as *Pfama1*, loci present with a combination of all three, wild type, mutant, and mixed in the population.

### Conclusions

This study shows, for the first time, candidate artemisinin resistance markers at a high frequency in asymptomatic infections, at a time prior to its detection in symptomatic infections, adding to the growing body of evidence of this malaria transmission reservoir harboring drug-resistant parasites that may facilitate their spread. The quintuple *Pfdhfr* and *Pfdhps* mutations remain at a high frequency, and *Pfmdr1* is 100% N86 in this population. Therefore, molecular surveillance is vital and should be sustained in view of reported clinical evidence of prolonged clearance of parasites harboring the C469Y mutation. Importantly, asymptomatic individuals should be considered during control efforts to reduce the prevalence and spread of drug resistance, particularly in high malaria transmission areas.

## MATERIALS AND METHODS

### Study design and setting

**Fig 2:**
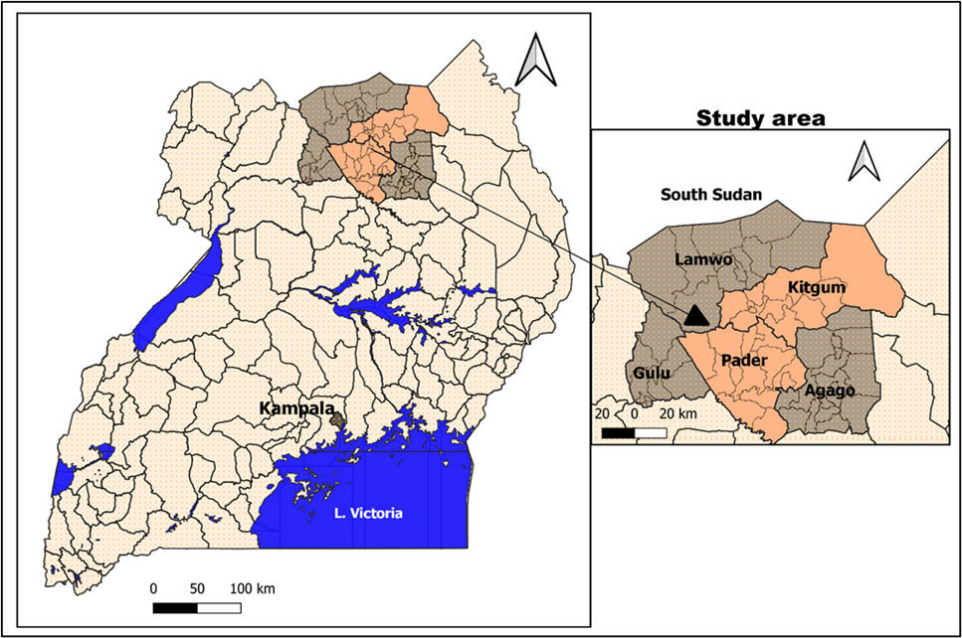
Map of Uganda showing the area where the samples were collected. The area in brick-red shows the districts of Kitgum and Pader in Northern Uganda, while the area in gray represents the surrounding district of Lamwo, Agago, and Gulu. The areas in blue shows water bodies. The maps were generated using QGIS.

This study was nested within a larger case control study investigating the pathogenesis of nodding syndrome—a poorly understood neurological disorder thought to be caused by an infection with the filarial worm *Onchocerca volvulus (44*). In the parent study, healthy individuals, 8 years or older, from the nodding syndrome-affected districts of Kitgum and Pader in Northern Uganda (Fig. 2), were recruited as healthy controls between 2016 and 2017 (44). In this area, inhabited by the Acholi people, malaria is a major cause of morbidity and mortality (24, 45). During the study period, the region was experiencing an upsurge in malaria cases, and a study from Kitgum general hospital found that malaria was the leading cause of hospitalization, accounting for 40% of inpatient admissions (24).

Between September 2016 and September 2017, 154 children and adolescents were enrolled as age-matched healthy controls into the parent study from within the surrounding communities. All enrolled study participants underwent a clinical examination with a detailed clinical history reported. A blood sample was collected from each participant, and dried blood spot prepared on Whatman filter paper. All were screened for malaria using *P. falciparum* HRP2 rapid diagnostic tests (CareStart, 2016). For this analysis, 142 samples collected from healthy community controls with no clinical symptoms were available.

### Laboratory procedures for DNA extraction, amplification, and sequencing

To extract parasite DNA from the RDT-positive DBS, a previously published protocol (12) was used. Briefly, two punches each 2.5 mm were punched from two locations (at the center and periphery) of the DBS and placed into a 1.5-mL Eppendorf tube using sterile tweezers. DNA extraction was done using the Chelex saponin method (46). Parasite DNA was amplified using 18S rRNA *Plasmodium falciparum* qPCR assay (47). Samples above the median cycling threshold of 35 were considered as low parasitemia, and they were excluded from sequencing. Using a previously published nested PCR approach (12), amplicons were generated in duplicates for the following genes, using molecular identifiers (MID) labeled primers (12) for *P. falciparum* apical membrane antigen 1 gene (*Pfama1*: PF3D7_1133400), *P. falciparum* dihydrofolate reductase (*Pfdhfr*: PF3D7_0417200), *P. falciparum* dihydropteroate synthase (*Pfdhps*: PF3D7_0810800), *P. falciparum* kelch13 (*Pfk13*: PF3D7_1347700), and *P. falciparum* multidrug resistance 1 (*Pfmdr1*: PF3D7_0523000). The ama1 primers spanned nucleotides 441–946, which overlap with domain I, a region with the highest nucleotide diversity in ama1 (28). The PCR products were individually purified using the AMPure XP beads (Beckman Coulter, Inc.) as per the manufacturer’s instructions. Thereafter, the quantification of the purified DNA was done using a Qubit double-strand DNA (dsDNA) High-Sensitivity (HS) Assay Kit, according to the manufacturer’s instructions. Library preparation was done using the KAPA Kit, while a size selection clean-up was done using 0.8× AMPure XP beads. The adapter-ligated libraries were then amplified using Illumina primers and cleaned with 0.8× AMPure. The libraries were quantified using a Qubit dsDNA HS Kit, and sizes were verified by the DNA 1000 Assay Kit using the 2100 Bioanalyzer (Agilent). The libraries were mixed in equimolar concentrations, denatured, spiked with 8% PhiX DNA, and finally sequenced using a MiSeq Reagent Kit v3 (Illumina).

### Sequence data analysis

SeekDeep v3.0.1 (48) was utilized for data analysis and involved three main algorithms for analyzing amplicon sequence data. The first algorithm, Extractor, performed demultiplexing of sequence data using the MIDs and primers utilized for amplification. Additionally, Extractor performed read filtering based on target-specific read lengths and trimmed bases with quality scores below 20. This ensured that only high-quality bases were included in subsequent analyses.

The second algorithm, Qluster, refined the microhaplotype identification process by collapsing amplicon reads by integrating sequencer-generated quality values and kmer frequencies. Amplicon sizes in the panel ranged from 500 to 520 base pairs, and microhaplotypes were constructed based on reads spanning these read lengths. Overlapping reads were stitched to ensure the continuity of the sequence data, contributing to accurate microhaplotype reconstruction. This approach effectively differentiated true biological variations from sequencing errors and PCR artifacts, thereby improving the reliability of microhaplotype inference. The clustering process involved an initial stage where reads with no differences were collapsed into identical sequence clusters indexed by their k-mers for efficient processing. This was followed by an iterative clustering stage, where clusters were compared iteratively with progressively stringent merging criteria, allowing for an adaptive response to the sequence diversity in the samples. Each mismatch was evaluated based on mismatch quality and the quality of adjacent bases, enhancing the accuracy of error distinction from true variants. To identify and mitigate PCR chimeras, sequences were examined for unusual patterns or breakpoints that typically indicate chimera formation. These chimeric sequences were flagged and excluded from the final microhaplotype assembly.

The third algorithm, process Clusters, further refined the microhaplotype identification by re-evaluating clusters for residual errors. This step involved re-processing clustered reads to remove any remaining low-frequency errors or artifacts, ensuring the final microhaplotype calls were highly accurate. Microhaplotypes were discarded if they did not occur in duplicate samples and if their combined relative frequency was <1%. A conservative cutoff of 1% was set based on the lowest lab isolate mixture (0.5:0.49:0.01; i.e., 50% 3D7, 49% HB3, 1% 7G8), unless the microhaplotype was independently detected in other samples at >1%. The SNP frequencies in the population were calculated as the number of samples that contained the SNP over the total number of samples genotyped to determine the total number of samples with wild-type mutant or mixed genotypes. For the microhaplotype frequencies, two approaches were used. The first is based on regarding each instance of a microhaplotype as an observation, determined by dividing the count of each microhaplotype’s occurrences by the total number of microhaplotype occurrences. The second approach used the within-host frequency of the haplotype as a weighting to adjust the haplotype frequency reported. All statistical analysis was performed in R v4.0.3 (49).

## Data Availability

The raw fastq files have been deposited in Harvard Dataverse: https://doi.org/10.7910/DVN/VGJYQW.

## SUPPLEMENTAL MATERIAL

Table S1 aac.01576-23-s0001.docx Weighted frequency of resistance haplotypes.

ASM does not own the copyrights to Supplemental Material that may be linked to, or accessed through, an article. The authors have granted ASM a non-exclusive, world-wide license to publish the Supplemental Material files. Please contact the corresponding author directly for reuse.

## References

[1] Rosenthal PJ. 2022. Malaria in 2022: challenges and progress. Am J Trop Med Hyg 106:1565–1567. doi:10.4269/ajtmh.22-0128 35413687 PMC9209912

[2] WHO. 2023. World malaria report 2023. World Health Organization

[3] Snow RW, Sartorius B, Kyalo D, Maina J, Amratia P, Mundia CW, Bejon P, Noor AM. 2017. The prevalence of Plasmodium falciparum in sub-Saharan Africa since 1900. Nature 550:515–518. doi:10.1038/nature24059 29019978 PMC5660624

[4] Noor AM, Alonso PL. 2022. The message on malaria is clear: progress has stalled. Lancet 399:1777. doi:10.1016/S0140-6736(22)00732-2 PMC9536759 35461617

[5] Ashley EA, Dhorda M, Fairhurst RM, Amaratunga C, Lim P, Suon S, Sreng S, Anderson JM, Mao S, Sam B, et al.. 2014. Spread of artemisinin resistance in Plasmodium falciparum malaria. N Engl J Med 371:411–423. doi:10.1056/NEJMoa1314981 25075834 PMC4143591

[6] Stokes BH, Ward KE, Fidock DA. 2022. Evidence of artemisinin-resistant malaria in Africa. N Engl J Med 386:1385–1386. doi:10.1056/NEJMc2117480 35388682 PMC9888016

[7] Rosenthal PJ. 2021. Has artemisinin resistance emerged in Africa? Lancet Infect Dis 21:1056–1057. doi:10.1016/S1473-3099(21)00168-7 33864802

[8] Nanyunja M, Nabyonga Orem J, Kato F, Kaggwa M, Katureebe C, Saweka J. 2011. Malaria treatment policy change and implementation: the case of Uganda. Malar Res Treat 2011:683167. doi:10.4061/2011/683167 22312571 PMC3265287

[9] Tse EG, Korsik M, Todd MH. 2019. The past, present and future of anti-malarial medicines. Malar J 18:93. doi:10.1186/s12936-019-2724-z 30902052 PMC6431062

[10] MoH U. 2016. Uganda clinical guidelines 2016 national guidelines for management of common conditions. In Health.

[11] Noviyanti R, Miotto O, Barry A, Marfurt J, Siegel S, Thuy-Nhien N, Quang HH, Anggraeni ND, Laihad F, Liu Y, Sumiwi ME, Trimarsanto H, Coutrier F, Fadila N, Ghanchi N, Johora FT, Puspitasari AM, Tavul L, Trianty L, Utami RAS, Wang D, Wangchuck K, Price RN, Auburn S. 2020. Implementing parasite genotyping into national surveillance frameworks: feedback from control programmes and researchers in the Asia–Pacific region. Malar J 19:271. doi:10.1186/s12936-020-03330-5 32718342 PMC7385952

[12] Osoti V, Akinyi M, Wamae K, Kimenyi KM, de Laurent Z, Ndwiga L, Gichuki P, Okoyo C, Kepha S, Mwandawiro C, Kandie R, Bejon P, Snow RW, Ochola-Oyier LI. 2022. Targeted amplicon deep sequencing for monitoring antimalarial resistance markers in Western Kenya. Antimicrob Agents Chemother 66:e0194521. doi:10.1128/aac.01945-21 35266823 PMC9017353

[13] Wamae K, Kimenyi KM, Osoti V, de Laurent ZR, Ndwiga L, Kharabora O, Hathaway NJ, Bailey JA, Juliano JJ, Bejon P, Ochola-Oyier LI. 2022. Amplicon sequencing as a potential surveillance tool for complexity of infection and drug resistance markers in Plasmodium falciparum asymptomatic infections. J Infect Dis 226:920–927. doi:10.1093/infdis/jiac144 35429395 PMC7613600

[14] Roux AT, Maharaj L, Oyegoke O, Akoniyon OP, Adeleke MA, Maharaj R, Okpeku M. 2021. Chloroquine and sulfadoxine–pyrimethamine resistance in sub-Saharan Africa—a review. Front Genet 12:12. doi:10.3389/fgene.2021.668574 PMC8267899 34249090

[15] Amaratunga C, Lim P, Suon S, Sreng S, Mao S, Sopha C, Sam B, Dek D, Try V, Amato R, Blessborn D, Song L, Tullo GS, Fay MP, Anderson JM, Tarning J, Fairhurst RM. 2016. Dihydroartemisinin–piperaquine resistance in Plasmodium falciparum malaria in Cambodia: a multisite prospective cohort study. Lancet Infect Dis 16:357–365. doi:10.1016/S1473-3099(15)00487-9 26774243 PMC4792715

[16] Noedl H, Se Y, Schaecher K, Smith BL, Socheat D, Fukuda MM, Artemisinin Resistance in Cambodia 1 (ARC1) Study Consortium. 2008. Evidence of artemisinin-resistant malaria in western Cambodia. N Engl J Med 359:2619–2620. doi:10.1056/NEJMc0805011 19064625

[17] Dondorp AM, Nosten F, Yi P, Das D, Phyo AP, Tarning J, Lwin KM, Ariey F, Hanpithakpong W, Lee SJ, Ringwald P, Silamut K, Imwong M, Chotivanich K, Lim P, Herdman T, An SS, Yeung S, Singhasivanon P, Day NPJ, Lindegardh N, Socheat D, White NJ. 2009. Artemisinin resistance in Plasmodium falciparum malaria. N Engl J Med 361:455–467. doi:10.1056/NEJMoa0808859 19641202 PMC3495232

[18] Balikagala B, Fukuda N, Ikeda M, Katuro OT, Tachibana S-I, Yamauchi M, Opio W, Emoto S, Anywar DA, Kimura E, Palacpac NMQ, Odongo-Aginya EI, Ogwang M, Horii T, Mita T. 2021. Evidence of artemisinin-resistant malaria in Africa. N Engl J Med 385:1163–1171. doi:10.1056/NEJMoa2101746 34551228

[19] Uwimana A, Umulisa N, Venkatesan M, Svigel SS, Zhou Z, Munyaneza T, Habimana RM, Rucogoza A, Moriarty LF, Sandford R, et al.. 2021. Association of Plasmodium falciparum kelch13 R561H genotypes with delayed parasite clearance in Rwanda: an open-label, single-arm, multicentre, therapeutic efficacy study. Lancet Infect Dis 21:1120–1128. doi:10.1016/S1473-3099(21)00142-0 33864801 PMC10202849

[20] Ikeda M, Kaneko M, Tachibana S-I, Balikagala B, Sakurai-Yatsushiro M, Yatsushiro S, Takahashi N, Yamauchi M, Sekihara M, Hashimoto M, Katuro OT, Olia A, Obwoya PS, Auma MA, Anywar DA, Odongo-Aginya EI, Okello-Onen J, Hirai M, Ohashi J, Palacpac NMQ, Kataoka M, Tsuboi T, Kimura E, Horii T, Mita T. 2018. Artemisinin-resistant Plasmodium falciparum with high survival rates, Uganda, 2014-2016. Emerg Infect Dis 24:718–726. doi:10.3201/eid2404.170141 29553316 PMC5875287

[21] Windle ST, Lane KD, Gadalla NB, Liu A, Mu J, Caleon RL, Rahman RS, Sá JM, Wellems TE. 2020. Evidence for linkage of pfmdr1, pfcrt, and pfk13 polymorphisms to lumefantrine and mefloquine susceptibilities in a Plasmodium falciparum cross. Int J Parasitol Drugs Drug Resist 14:208–217. doi:10.1016/j.ijpddr.2020.10.009 33197753 PMC7677662

[22] Ariey F, Witkowski B, Amaratunga C, Beghain J, Langlois A-C, Khim N, Kim S, Duru V, Bouchier C, Ma L, et al.. 2014. A molecular marker of artemisinin-resistant Plasmodium falciparum malaria. Nature 505:50–55. doi:10.1038/nature12876 24352242 PMC5007947

[23] Amambua-Ngwa A, Amenga-Etego L, Kamau E, Amato R, Ghansah A, Golassa L, Randrianarivelojosia M, Ishengoma D, Apinjoh T, Maïga-Ascofaré O, Andagalu B, Yavo W, Bouyou-Akotet M, Kolapo O, Mane K, Worwui A, Jeffries D, Simpson V, D’Alessandro U, Kwiatkowski D, Djimde AA. 2019. Major subpopulations of Plasmodium falciparum in sub-Saharan Africa. Science 365:813–816. doi:10.1126/science.aav5427 31439796

[24] Ogwang R, Akena G, Yeka A, Osier F, Idro R. 2018. The 2015-2016 malaria epidemic in Northern Uganda; what are the implications for malaria control interventions? Acta Trop 188:27–33. doi:10.1016/j.actatropica.2018.08.023 30145260 PMC7116666

[25] Drakeley C. 2018. Understanding the importance of asymptomatic and low-density infections for malaria elimination, p 1–20. In Towards malaria elimination-a leap forward.

[26] Kimenyi KM, Wamae K, Ochola-Oyier LI. 2019. Understanding P. falciparum asymptomatic infections: a proposition for a transcriptomic approach. Front Immunol 10:2398. doi:10.3389/fimmu.2019.02398 31681289 PMC6803459

[27] Galatas B, Bassat Q, Mayor A. 2016. Malaria parasites in the asymptomatic: looking for the hay in the haystack. Trends Parasitol. 32:296–308. doi:10.1016/j.pt.2015.11.015 26708404

[28] Polley SD, Conway DJ. 2001. Strong diversifying selection on domains of the Plasmodium falciparum apical membrane antigen 1 gene. Genetics 158:1505–1512. doi:10.1093/genetics/158.4.1505 11514442 PMC1461755

[29] Tumwebaze P, Tukwasibwe S, Taylor A, Conrad M, Ruhamyankaka E, Asua V, Walakira A, Nankabirwa J, Yeka A, Staedke SG, Greenhouse B, Nsobya SL, Kamya MR, Dorsey G, Rosenthal PJ. 2017. Changing antimalarial drug resistance patterns identified by surveillance at three sites in Uganda. J Infect Dis 215:631–635. doi:10.1093/infdis/jiw614 28039354 PMC5853976

[30] Asua V, Vinden J, Conrad MD, Legac J, Kigozi SP, Kamya MR, Dorsey G, Nsobya SL, Rosenthal PJ. 2019. Changing molecular markers of antimalarial drug sensitivity across Uganda. Antimicrob Agents Chemother 63:e01818–18. doi:10.1128/AAC.01818-18 30559133 PMC6395896

[31] Rogerson SJ, Desai M, Mayor A, Sicuri E, Taylor SM, van Eijk AM. 2018. Burden, pathology, and costs of malaria in pregnancy: new developments for an old problem. Lancet Infect Dis 18:e107–e118. doi:10.1016/S1473-3099(18)30066-5 29396010

[32] Kamau A, Musau M, Mwakio S, Amadi D, Nyaguara A, Bejon P, Seale AC, Berkley JA, Snow RW. 2023. Impact of intermittent presumptive treatment for malaria in pregnancy on hospital birth outcomes on the Kenyan coast. Clin Infect Dis 76:e875–e883. doi:10.1093/cid/ciac509 35731850 PMC9907553

[33] Asua V, Conrad MD, Aydemir O, Duvalsaint M, Legac J, Duarte E, Tumwebaze P, Chin DM, Cooper RA, Yeka A, Kamya MR, Dorsey G, Nsobya SL, Bailey J, Rosenthal PJ. 2021. Changing prevalence of potential mediators of aminoquinoline, antifolate, and artemisinin resistance across Uganda. J Infect Dis 223:985–994. doi:10.1093/infdis/jiaa687 33146722 PMC8006419

[34] Conrad MD, Asua V, Garg S, Giesbrecht D, Niaré K, Smith S, Namuganga JF, Katairo T, Legac J, Crudale RM, Tumwebaze PK, Nsobya SL, Cooper RA, Kamya MR, Dorsey G, Bailey JA, Rosenthal PJ. 2023. Evolution of partial resistance to artemisinins in malaria parasites in Uganda. N Engl J Med 389:722–732. doi:10.1056/NEJMoa2211803 37611122 PMC10513755

[35] Tumwebaze PK, Conrad MD, Okitwi M, Orena S, Byaruhanga O, Katairo T, Legac J, Garg S, Giesbrecht D, Smith SR, Ceja FG, Nsobya SL, Bailey JA, Cooper RA, Rosenthal PJ. 2022. Decreased susceptibility of Plasmodium falciparum to both dihydroartemisinin and lumefantrine in northern Uganda. Nat Commun 13:6353. doi:10.1038/s41467-022-33873-x 36289202 PMC9605985

[36] Okullo AE, Matovu JKB, Ario AR, Opigo J, Wanzira H, Oguttu DW, Kalyango JN. 2017. Malaria incidence among children less than 5years during and after cessation of indoor residual spraying in Northern Uganda. Malar J 16:319. doi:10.1186/s12936-017-1966-x 28784119 PMC5547524

[37] Tukei BB, Beke A, Lamadrid-Figueroa H. 2017. Assessing the effect of indoor residual spraying (IRS) on malaria morbidity in Northern Uganda: a before and after study. Malar J 16:4. doi:10.1186/s12936-016-1652-4 28049475 PMC5209922

[38] Nyunt MH, Shein T, Zaw NN, Han SS, Muh F, Lee S-K, Han J-H, Thant KZ, Han E-T, Kyaw MP. 2017. Molecular evidence of drug resistance in asymptomatic malaria infections, Myanmar, 2015. Emerg Infect Dis 23:517–520. doi:10.3201/eid2303.161363 28221121 PMC5382746

[39] Amaratunga C. 2019. Association of mutations in the Plasmodium falciparum kelch13 gene (Pf3D7_1343700) with parasite clearance rates after artemisinin-based treatments—a WWARN individual patient data meta-analysis. BMC Med 17:1. doi:10.1186/s12916-018-1207-3 30651111 PMC6335805

[40] Mahdi Abdel Hamid M, Elamin AF, Albsheer MMA, Abdalla AAA, Mahgoub NS, Mustafa SO, Muneer MS, Amin M. 2016. Multiplicity of infection and genetic diversity of Plasmodium falciparum isolates from patients with uncomplicated and severe malaria in Gezira State, Sudan. Parasites Vectors 9:362. doi:10.1186/s13071-016-1641-z 27350250 PMC4924276

[41] Ndwiga L, Kimenyi KM, Wamae K, Osoti V, Akinyi M, Omedo I, Ishengoma DS, Duah-Quashie N, Andagalu B, Ghansah A, Amambua-Ngwa A, Tukwasibwe S, Tessema SK, Karema C, Djimde AA, Dondorp AM, Raman J, Snow RW, Bejon P, Ochola-Oyier LI. 2021. A review of the frequencies of Plasmodium falciparum kelch 13 artemisinin resistance mutations in Africa. Int J Parasitol Drugs Drug Resist 16:155–161. doi:10.1016/j.ijpddr.2021.06.001 34146993 PMC8219943

[42] Muhindo Mavoko H, Kalabuanga M, Delgado-Ratto C, Maketa V, Mukele R, Fungula B, Inocêncio da Luz R, Rosanas-Urgell A, Lutumba P, Van Geertruyden J-P. 2016. Uncomplicated clinical malaria features, the efficacy of artesunate-amodiaquine and their relation with multiplicity of infection in the democratic republic of Congo. PLoS One 11:e0157074. doi:10.1371/journal.pone.0157074 27280792 PMC4900589

[43] Miller RH, Hathaway NJ, Kharabora O, Mwandagalirwa K, Tshefu A, Meshnick SR, Taylor SM, Juliano JJ, Stewart VA, Bailey JA. 2017. A deep sequencing approach to estimate Plasmodium falciparum complexity of infection (COI) and explore apical membrane antigen 1 diversity. Malar J 16:490. doi:10.1186/s12936-017-2137-9 29246158 PMC5732508

[44] Ogwang R, Muhanguzi D, Mwikali K, Anguzu R, Kubofcik J, Nutman TB, Taylor M, Newton CR, Vincent A, Conroy AL, Marsh K, Idro R. 2021. Systemic and cerebrospinal fluid immune and complement activation in Ugandan children and adolescents with long-standing nodding syndrome: a case-control study. Epilepsia Open 6:297–309. doi:10.1002/epi4.12463 34033255 PMC8166803

[45] Kigozi SP, Kigozi RN, Sebuguzi CM, Cano J, Rutazaana D, Opigo J, Bousema T, Yeka A, Gasasira A, Sartorius B, Pullan RL. 2020. Spatial-temporal patterns of malaria incidence in Uganda using HMIS data from 2015 to 2019. BMC Public Health 20:1913. doi:10.1186/s12889-020-10007-w 33317487 PMC7737387

[46] Baidjoe A, Stone W, Ploemen I, Shagari S, Grignard L, Osoti V, Makori E, Stevenson J, Kariuki S, Sutherland C, Sauerwein R, Cox J, Drakeley C, Bousema T. 2013. Combined DNA extraction and antibody elution from filter papers for the assessment of malaria transmission intensity in epidemiological studies. Malar J 12:272. doi:10.1186/1475-2875-12-272 23914905 PMC3750228

[47] Hermsen CC, Telgt DS, Linders EH, van de Locht LA, Eling WM, Mensink EJ, Sauerwein RW. 2001. Detection of Plasmodium falciparum malaria parasites in vivo by real-time quantitative PCR. Mol Biochem Parasitol 118:247–251. doi:10.1016/s0166-6851(01)00379-6 11738714

[48] Hathaway NJ, Parobek CM, Juliano JJ, Bailey JA. 2018. SeekDeep: single-base resolution de novo clustering for amplicon deep sequencing. Nucleic Acids Res 46:e21. doi:10.1093/nar/gkx1201 29202193 PMC5829576

[49] R Core Team. 2022. R: a language and environment for statistical computing. R foundation for statistical computing, Vienna. Available from: https://www.R-project.org

