# Supplementary material for: A retrospective analysis of P. falciparum drug resistance markers detects an early (2016/17) high prevalence of the k13 C469Y mutation in asymptomatic infections in Northern Uganda
Source: Antimicrob Agents Chemother. 2024 Aug 13;68(9):e01576-23. doi: 10.1128/aac.01576-23 (PMC11382623; doi:10.1128/aac.01576-23)
Supplement: Table S1 — Weighted frequency of resistance haplotypes. [file aac.01576-23-s0001.docx]

**Supplementary Table S1. Weighted frequency of resistance haplotypes for each gene and of infections containing mixed haplotypes**

| **Gene**  **(Codon)** | **Haplotype** | **n** | **Frequency (%)** |
| --- | --- | --- | --- |
| *dhfr* | **IRN**I | 58 | 87.6 |
|  | **I**C**N**I | 37 | 11.4 |
| (51,59,108,164) | N**RN**I | 9 | 0.2 |
|  | **IRNL** | 8 | 0.7 |
|  | NCSI* | 1 | 0.1 |
| *dhps* | S**GE**A | 71 | 81.9 |
|  | S**G**KA | 61 | 6.7 |
| (436,437,540,581) | SAKA* | 27 | 6 |
|  | SA**E**A | 24 | 3.2 |
|  | S**GEG** | 7 | 0.3 |
|  | **C**A**E**A | 3 | 0.4 |
|  | **C**AKA | 3 | 0.9 |
|  | **A**A**E**A | 2 | 0.4 |
| *k13* | CYEA* | 50 | 92.5 |
| (469,482,507,578) | **Y**YEA | 17 | 7.1 |
|  | CYE**S** | 2 | * |
|  | C**H**EA | 2 | 0.1 |
|  | **Y**Y**G**A | 2 | 0.3 |
| *mdr1* | N**F**T | 67 | 59.1 |
| (86,184,199) | NYT* | 64 | 36.7 |
|  | NY**S** | 21 | 4.2 |
